# A novel somatic mutation in *GNB2* provides new insights to the pathogenesis of Sturge–Weber syndrome

**DOI:** 10.1093/hmg/ddab144

**Published:** 2021-06-14

**Authors:** Roar Fjær, Katarzyna Marciniak, Olav Sundnes, Hanne Hjorthaug, Ying Sheng, Clara Hammarström, Jan Cezary Sitek, Magnus Dehli Vigeland, Paul Hoff Backe, Ane-Marte Øye, Johanna Hol Fosse, Tor Espen Stav-Noraas, Yuri Uchiyama, Naomichi Matsumoto, Anne Comi, Jonathan Pevsner, Guttorm Haraldsen, Kaja Kristine Selmer

**Affiliations:** Department of Medical Genetics, Oslo University Hospital and University of Oslo, Oslo 0424, Norway; Department of Neurology and Clinical Neurophysiology, St. Olavs University Hospital, Trondheim 3250, Norway; Institute of Clinical Medicine, University of Oslo, Oslo 0424, Norway; K.G. Jebsen Inflammation Research Centre, University of Oslo, Oslo 0424, Norway; Department of Pathology, Oslo University Hospital, Oslo 0424, Norway; K.G. Jebsen Inflammation Research Centre, University of Oslo, Oslo 0424, Norway; Department of Pathology, Oslo University Hospital, Oslo 0424, Norway; Department of Dermatology, Oslo University Hospital, Oslo 0424, Norway; Department of Medical Genetics, Oslo University Hospital and University of Oslo, Oslo 0424, Norway; Department of Medical Genetics, Oslo University Hospital and University of Oslo, Oslo 0424, Norway; K.G. Jebsen Inflammation Research Centre, University of Oslo, Oslo 0424, Norway; Department of Pathology, Oslo University Hospital, Oslo 0424, Norway; Department of Dermatology, Oslo University Hospital, Oslo 0424, Norway; Department of Medical Genetics, Oslo University Hospital and University of Oslo, Oslo 0424, Norway; Institute of Clinical Medicine, University of Oslo, Oslo 0424, Norway; Department of Microbiology, Oslo University Hospital, Oslo 0424, Norway; Department of Medical Biochemistry, Oslo University Hospital, Oslo 0424, Norway; Department of Medical Genetics, Oslo University Hospital and University of Oslo, Oslo 0424, Norway; K.G. Jebsen Inflammation Research Centre, University of Oslo, Oslo 0424, Norway; Department of Pathology, Oslo University Hospital, Oslo 0424, Norway; K.G. Jebsen Inflammation Research Centre, University of Oslo, Oslo 0424, Norway; Department of Human Genetics, Yokohama City University Graduate School of Medicine, Yokohama 236-0004, Japan; Department of Rare Disease Genomics, Yokohama City University Hospital, Yokohama 236-0004, Japan; Department of Human Genetics, Yokohama City University Graduate School of Medicine, Yokohama 236-0004, Japan; Department of Neurology, Hugo Moser Kennedy Krieger Research Institute, Baltimore, MD 21205, USA; Department of Neurology, Johns Hopkins University School of Medicine, Baltimore, MD 21205, USA; Department of Pediatrics, Johns Hopkins University School of Medicine, Baltimore, MD 21205, USA; Department of Neurology, Hugo Moser Kennedy Krieger Research Institute, Baltimore, MD 21205, USA; Department of Psychiatry and Behavioral Sciences, John Hopkins University School of Medicine, Baltimore, MD 21205, USA; K.G. Jebsen Inflammation Research Centre, University of Oslo, Oslo 0424, Norway; Department of Pathology, Oslo University Hospital, Oslo 0424, Norway; Department of Medical Genetics, Oslo University Hospital and University of Oslo, Oslo 0424, Norway; National Centre for Rare Epilepsy-related Disorders, Oslo University Hospital and the University of Oslo, Oslo 0424, Norway; Department of Research and Innovation, Division of Clinical Neuroscience, Oslo University Hospital, Oslo 0424, Norway

## Abstract

Sturge–Weber syndrome (SWS) is a neurocutaneous disorder characterized by vascular malformations affecting skin, eyes and leptomeninges of the brain, which can lead to glaucoma, seizures and intellectual disability. The discovery of a disease-causing somatic missense mutation in the *GNAQ* gene, encoding an alpha chain of heterotrimeric G-proteins, has initiated efforts to understand how G-proteins contribute to SWS pathogenesis. The mutation is predominantly detected in endothelial cells and is currently believed to affect downstream MAPK signalling. In this study of six Norwegian patients with classical SWS, we aimed to identify somatic mutations through deep sequencing of DNA from skin biopsies. Surprisingly, one patient was negative for the *GNAQ* mutation, but instead harbored a somatic mutation in *GNB2* (NM_005273.3:c.232A>G, p.Lys78Glu), which encodes a beta chain of the same G-protein complex. The positions of the mutant amino acids in the G-protein are essential for complex reassembly. Therefore, failure of reassembly and continuous signalling is a likely consequence of both mutations. Ectopic expression of mutant proteins in endothelial cells revealed that expression of either mutant reduced cellular proliferation, yet regulated MAPK signalling differently, suggesting that dysregulated MAPK signalling cannot fully explain the SWS phenotype. Instead, both mutants reduced synthesis of Yes-associated protein (YAP), a transcriptional co-activator of the Hippo signalling pathway, suggesting a key role for this pathway in the vascular pathogenesis of SWS. The discovery of the *GNB2* mutation sheds novel light on the pathogenesis of SWS and suggests that future research on targets of treatment should be directed towards the YAP, rather than the MAPK, signalling pathway.

## Introduction

Sturge–Weber syndrome [Mendelian Inheritance in Man (MIM): 185300] is a rare neurocutaneous disorder of dilated post-capillary venules in the eye, skin and the leptomeninges of the brain. Due to its well-circumscribed borders, absence of systemic symptoms and a generally unilateral location, it has been believed to be caused by a somatic mutation (1). This hypothesis was confirmed in 2013 by Shirley *et al*. (2), who found a single somatic missense mutation in the *GNAQ* gene (MIM: 600998) in 23 out of 26 patients with Sturge–Weber syndrome and in 12 out of 13 patients with port-wine birthmarks. Additional studies have confirmed that the somatic mutation in *GNAQ* is found in affected tissue in about 70–90% of the patients with Sturge–Weber syndrome and port-wine birthmarks (3–8).

*GNAQ* encodes a G-protein alpha subunit (Gαq) of heterotrimeric G-proteins, and the mutation reported in Sturge–Weber syndrome is considered to be an activating mutation (NM_002072.5: c.548G>A, p.Arg183Gln, the protein variant will from here onward be referred to as GNAQ R183Q), where intrinsic GTPase activity increases downstream signalling. This finding is now shaping a novel understanding of the molecular cues that lead to Sturge–Weber syndrome and other vascular malformations. A longstanding hypothesis has been that Sturge–Weber syndrome results from aberrant early vascular development, due to a somatic mutation originating in a subset of angioblasts (9). Support for this assumption was found in a study by Couto *et al.* who sequenced DNA from purified dermal endothelial cells from patients with sporadic capillary malformations and revealed that 10 out of 13 samples harboured the c.548G>A mutation in *GNAQ* (3). Other studies have later corroborated this finding in brain endothelial cells (6).

The heterotrimeric G-protein complex consists of three subunits, the alpha, the beta and the gamma subunit. It is intracellularly located, associated with a G-protein-coupled receptor (GPCR) and responsible for transmitting extracellular signals. In an active state, the protein complex disassociates to an alpha unit and a beta-gamma dimer, which both affect several downstream signalling pathways. The main hypothesis concerning the functional impact of the *GNAQ* mutation in Sturge–Weber syndrome has been that the decreased GTPase activity results in a moderate increase in mitogen-activated protein kinase (MAPK) signalling (2). Emerging data from cancer research have also raised the possibility that the transcriptional co-activator YAP, a central mediator of the Hippo pathway, may be involved in a Hippo-independent manner (10,11), and the current understanding of the pathogenesis is that the *GNAQ* mutation causes excessive activation of several downstream pathways including MAPK and YAP (12).

In this study, we aimed to identify, characterize and localize the cellular origin of the genetic cause of Sturge–Weber syndrome in a cohort of Norwegian patients and to explore the underlying mechanisms of disease development. In our cohort, we found that five out of six patients harboured the *GNAQ* mutation and that it was enriched in endothelial cells. Intriguingly, in the *GNAQ* mutation-negative patient, we discovered a novel somatic mutation in *GNB2* (MIM: 139390), which encodes a β subunit of the heterotrimeric G-protein complex. Importantly, this mutation was also enriched in endothelial cells and provides an alternative molecular basis for aberrant G-protein signalling in Sturge–Weber syndrome lesions. Indeed, ectopic expression of mutant and wild-type *GNAQ* and *GNB2* induced differential MAPK phosphorylation, but similar changes in the YAP pathway, indicating that the latter pathway may be more relevant to the development of Sturge–Weber syndrome than the MAPK pathway.

## Results

### Clinical and imaging characteristics of the patients

**Figure 1:**
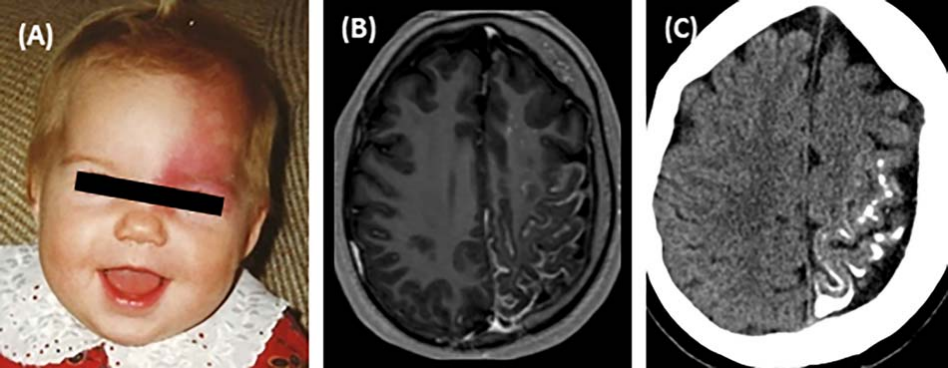
Clinical features of patient 3. (**A**) Childhood image of the patient displaying a port-wine lesion affecting her left eyelid and brow. (**B**) Contrast-enhanced MRI shows left-sided enhancement in leptomeningeal vascular malformation and atrophy. (**C**) Non-contrast-enhanced CT shows gyral calcifications and atrophy.

The clinical characteristics of the six enrolled patients are summarized in Table 1. Briefly, all had congenital facial port-wine birthmarks, three had eye involvement and all had findings compatible with vascular malformations on brain MRI. In particular, patient 3 with the *GNB2* mutation had a classic port-wine birthmark affecting her left eye and brow, and her MRI and CT images demonstrated classic Sturge–Weber syndrome-associated features (Fig. 1).

**Table 1:** Clinical characteristics of patients, and locations of biopsies

Patient	Age	Skin lesions	Cognitive function	Epilepsy onset (seizure type)	Epilepsy status (ASM)	MRI findings	Eye affection	Lesional biopsy	Non-lesional biopsy
1	25	Unilateral left brow and temple	Normal, but learning difficulties	7 months (focal seizures)	Medically controlled (lamotrigine)	Slight atrophy in the posterior left parietal lobe and occipital lobe. T2-weighted MRI with low signal representing calcification in the same localization. Enlarged deep draining veins left side	No	Below hairline, left brow	Medial left upper arm
2	27	Face and extremities right side, scattered changes in right hemithorax	Mild intellectual disability	1 year (bilateral tonic clonic seizures)	Medically controlled (carbamazepine)	Right-sided atrophy, dark subcortical deposits representing calcifications (non-contrast MRI) increased soft tissue volume face and orbitae	Glaucoma	Right upper arm	Unaffected area of right upper arm
3	22	Unilateral left eyelid, nose and brow	Mild intellectual disability	Not known	Refractory (oxcarbazepine)	Left-sided leptomeningeal vascular malformation and atrophy	No	Below hairline, left brow	Medial left upper arm
4	12	Right brow and eyelid	Normal	3 months	Undergone resective epilepsy surgery, some focal seizures with impairment of consciousness (carbamazepine)	Right-sided frontoparietal leptomeningeal vascular malformation. Slight atrophy of right hemisphere (presurgical MRI)	No	Below hairline, right brow	Medial left upper arm
5	21	Face upper right side, scattered changes both sides of the body	Moderate intellectual disability	3 months (focal seizures with generalization)	Not known	Not known	Glaucoma	Right scapular region	Left scapular region
6	60	Left side of face, scattered trunk and limbs lesions	Normal	40 years (focal seizures)	Intermittent focal seizures (4/year), unmedicated	Left parieto-occipital vascular malformation with slight atrophy. Age-related changes	Congenital glaucoma	Left of sternum	Medial right upper arm

ASM, anti-seizure medication, MRI, magnetic resonance imaging.

### Targeted sequencing of *GNAQ*:c.548G>A

Samples from affected and unaffected dermis (patients 2–6), available cell cultures of endothelial cells, keratinocytes and fibroblasts (patients 1–5), as well as laser capture microdissected material (patients 2 and 6) and a mutation positive control sample were subject to targeted amplicon sequencing (with MiSeq) covering the *GNAQ*:c.548G>A variant (selected samples Table 2, all samples are shown in Supplementary Material, Table S1). Five out of six affected dermal biopsies were positive for the *GNAQ*:c.548G>A mutation. Endothelial cell cultures from three out of the four patient samples that could be analysed were positive for *GNAQ*:c.548G>A, with mutation frequencies ranging from 0.2 to 14%. All keratinocyte and fibroblast cultures were negative for the mutation. Samples with laser capture microdissected endothelium from patients 2 and 6 were subsequently sequenced, resulting in higher ratios of *GNAQ*:c.548G>A mutations, 30 and 20%, respectively.

**Table 2:** Synopsis of pertinent MiSeq sequencing of GNAQ:c.548G>A results from all affected samples

Patient	Sample	Mutation frequency (%)	Depth	G (ref)	A (mut)	T	C
1	Endothelial cell culture	14.36	130 299	111 585	18 709	4	1
2	Dermis	7.08	128 341	119 233	9091	16	1
	Endothelial cell culture	9.28	100 355	91 036	9314	5	0
	LCM endothelium	29.88	60 684	42 552	18 131	1	0
3	Endothelial cell culture	0.01	99 182	99 159	14	6	3
	Dermis	0.01	131 325	131 300	16	8	1
4	Dermis	7.39	135 365	125 357	9998	8	2
	Endothelial cell culture 1	0.24	113 991	113 714	269	5	3
	Endothelial cell culture 2	0.13	143 019	142 825	189	4	1
5	Dermis	8.98	147 823	134 533	13 276	12	2
6	Dermis	5.72	99 490	93 794	5694	0	2
	LCM endothelium	19.93	192 942	154 496	38 444	0	2

ref, reference allele; mut, mutation, LCM, laser capture microdissection.

### Whole-exome sequencing of the *GNAQ* mutation-negative patient

Deep whole-exome sequencing of DNA from lesional dermis of patient 3 revealed two candidate variants. One candidate was the *GNB2* (NM_005273.3):c.232A>G:p.Lys78Glu, which was present in 6% (34/564) of the reads. MiSeq validation of the variant in the same sample was positive in 3.6% (3265/91 041) of the reads, and positive in 21% (14 858/71 630) of the reads in the endothelial culture from the biopsy, but only present at 0.15% (206/133 486) of the reads in non-lesional dermis (Table 3). Sequencing thus revealed a considerably larger ratio of mutation-positive DNA in the endothelial culture than in the dermis. Another candidate variant from the exome data (*ZNF518B* (NM_053042):c.A641C:p.Glu214Ala) failed MiSeq validation. MiSeq deep sequencing of all exons and exon–intron boundaries of *GNB2* and the homolog *GNB1* in DNA from brain from five additional *GNAQ* mutation-negative patients was performed at the Kennedy Krieger Institute, but none were found to harbour mutations. Likewise, targeted amplicon sequencing with MiSeq of the coding sequences of *GNB2* in DNA from blood and brain tissue from a Japanese *GNAQ* mutation-negative patient did not reveal any somatic mutations.

**Table 3:** MiSeq results of GNB2:c.232A>G in samples from patient 3

Patient 3	Mutation frequency (%)	Depth	A (ref)	G (mut)	T	C
Affected dermis	3.59	91 041	87 769	3265	7	0
Affected endothelial cell culture	20.74	71 630	56 771	14 858	1	0
Affected fibroblast cell culture	0.02	112 155	112 128	19	8	0
Affected keratinocyte cell culture	0.01	129 481	129 464	13	4	0
Unaffected dermis	0.15	133 486	133 264	206	15	1
Unaffected fibroblast cell culture	0.01	53 404	53 400	3	1	0

ref, reference allele; mut, mutation.

### Structural analysis indicates that the GNB2 mutation disrupts a salt bridge bond between the Gα subunit and the Gβϒ subunit

**Figure 2:**
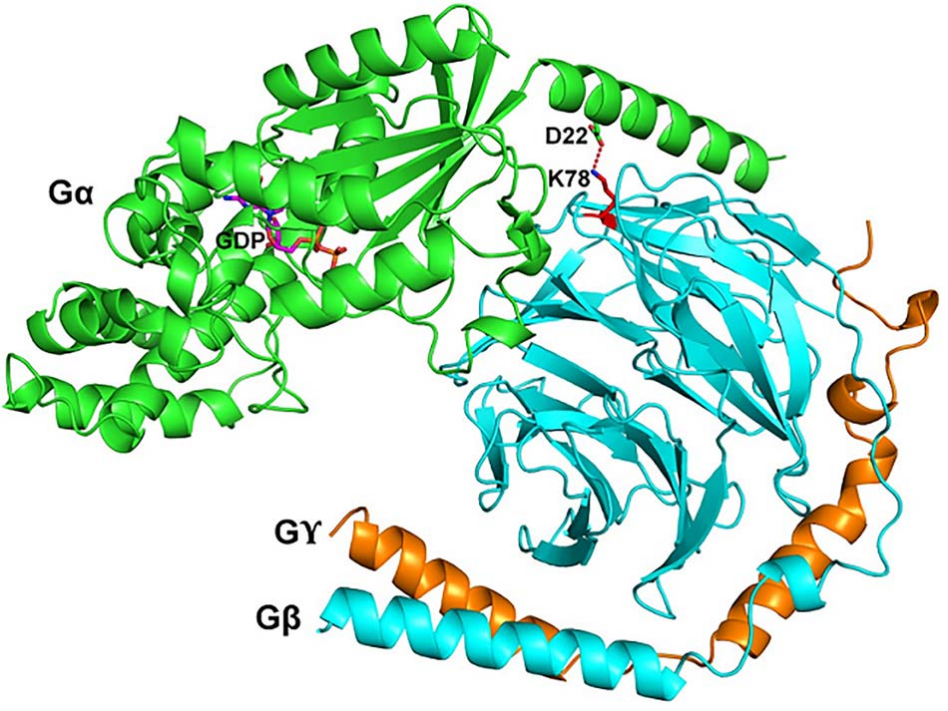
Cartoon representation of a heterotrimeric G-protein complex showing the location of the GNB2 K78 position. The alpha q subunit (Gα) shown in green, the beta subunit (Gβ) in cyan, the gamma subunit (Gϒ) in orange and GDP in magenta. The model is based on the 2.0 Å crystal structure of a heterotrimeric G-protein (PDB ID: 1GOT) (23). The GNB2 K78 (depicted in red) is located in the β-propeller of the Gβ subunit and interacts with D22 in N-terminal helix of Gα through a salt bridge. The figure was made with PYMOL (http://www.pymol.org).

The identified *GNB2*:c.232A>G mutation causes a substitution of lysine to glutamic acid at position 78 in the GNB2 protein (protein variant from here onward referred to as GNB2 K78E). To make structural assumptions and assess possible functional consequences of the K78E mutation, we took advantage of the known crystal structure describing subunit interactions of the modified heterotrimeric complex of transducin (13). In a ribbon diagram of the transducin structure (Fig. 2), lysine at position 78 of GNB1 (corresponding to the same position in GNB2) is located in the β-propeller domain of Gβ and faces the N-terminal helix of GNAT1 where it forms an intermolecular salt bridge with the aspartic acid at position 22 (corresponding to Asp32 in GNAQ) in the N-terminal helix of Gα (Fig. 2). We may then speculate that if lysine at position 78 of GNB2 is mutated to glutamic acid, cationic ammonium from Lys78 is lost and disrupts the salt bridge to the anionic carboxylate of Asp32, instead inducing a charge repulsion and impairing binding to the alpha chain. The novel somatic mutation identified in *GNB2* K78E may therefore provide an alternative molecular basis for increased Gαq signalling.

### GNAQ R183Q and GNB2 K78E reduce proliferation, but differentially affect endothelial cell migration

**Figure 3:**
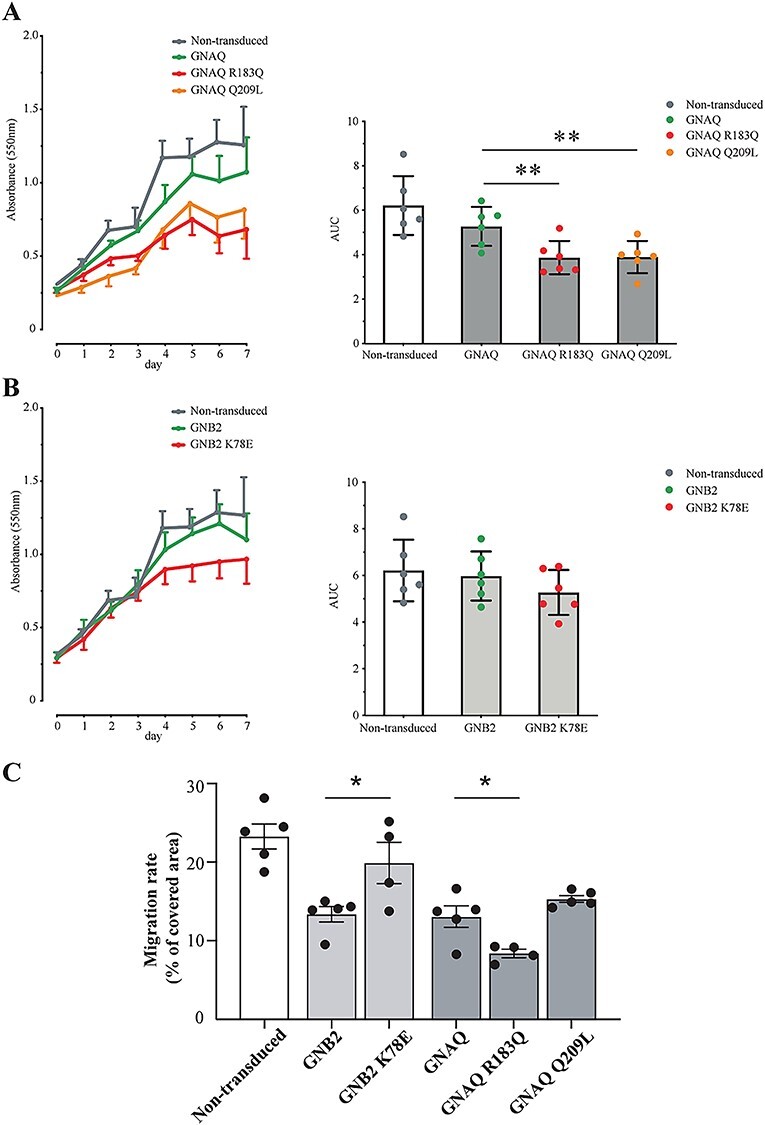
Mutations in *GNAQ* and *GNB2* reduce proliferation, but differentially affect migration. Proliferation curves for non-transduced (grey curves) and adenovirus-transduced HUVECs showing in (**A**) wild-type GNAQ (green curve), GNAQ R183Q (red curve) and GNAQ Q209L (orange curve), and in (**B**) wild-type GNB2 (green curve) and GNB2 K78E (red curve). Bars in panels right show mean ± standard error of the mean (SEM) of areas under curves (AUC) with individual data points showing mean values from six independent experiments, each containing three technical replicates. ^**^P < 0.01. (**C**) Migration of HUVECs transduced with adenovirus vectors. Scratched areas in cell monolayers were measured at the moment of generating the scratch (0 h) and after 6 h. Bars show mean% ± standard deviation of covered area of the gap, with individual data points showing mean values from four or five independent experiments, each containing 36 technical replicates. ^*^P < 0.05. All *P*-values are listed in Supplementary Material, Table S2.

The vascular malformation of the Sturge–Weber syndrome lesion is one of dilated venules, revealing a dysregulated pattern of vascular morphogenesis and likely involving altered proliferation and/or migration of endothelial cells (14). To test whether the GNAQ R183Q and GNB2 K78E mutation may affect endothelial cell behaviour in a similar way, we next assessed how ectopic expression of mutants affected proliferation of human umbilical vein endothelial cells (HUVECs), using non-transduced cells and wild-type protein as controls. Adenoviral transduction of vectors encoding wtGNAQ, GNAQ R183Q, *GNAQ*:c626A>T (p.Gln209Leu, protein variant from here on referred to as GNAQ Q209L), wtGNB2 and GNB2 K78E revealed that all mutations showed reduced or a trend towards reduced proliferation rates compared to their corresponding wild-type control (Fig. 3A and B, all *P*-values in Supplementary Material, Table S2). We next assessed how mutants affected the endothelial cell migration rate by performing scratch assays. Surprisingly, these analyses revealed that while the GNAQ R183Q mutation seen in the majority of Sturge–Weber syndrome patients reduced migration compared to wtGNAQ, GNB2 K78E enhanced migration relative to controls (Fig. 3C). The GNAQ Q209L also showed a trend towards enhanced migration (Fig. 3C).

### GNAQ R183Q and GNB2 K78E reduce YAP levels while differentially affecting MAPK phosphorylation

**Figure 4:**
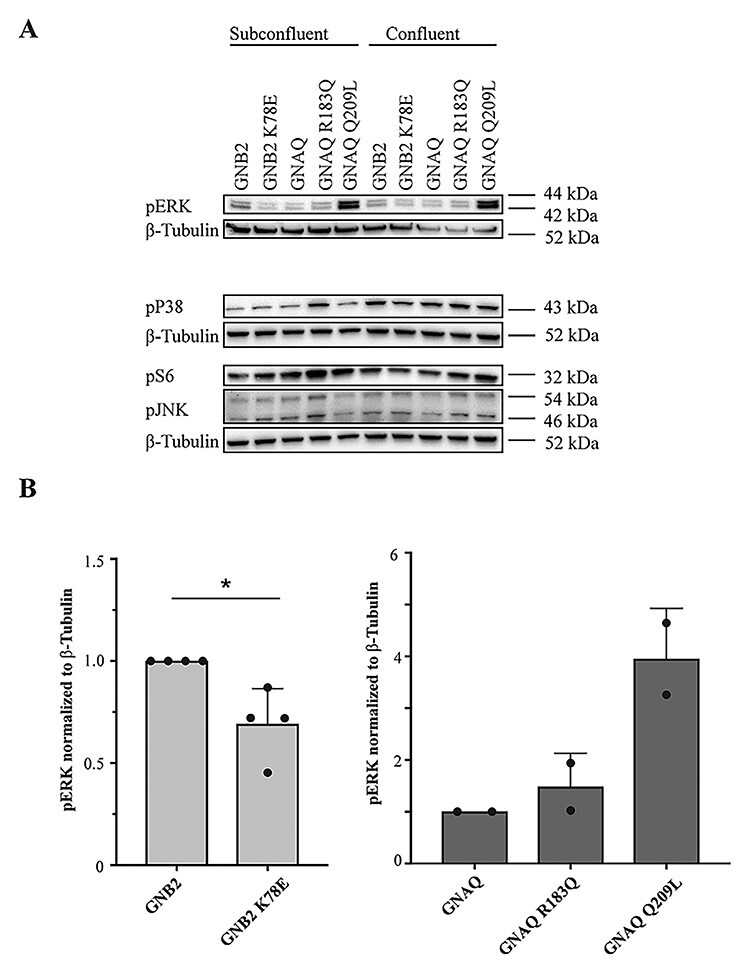
Mutations in *GNAQ* and *GNB2* differentially affect MAPK phosphorylation. Western blot of lysates of confluent and subconfluent HUVECs transduced with adenovirus vectors as indicated by labels. (**A**) Representative blots incubated with antibodies to pERK, pP38, pS6, pJNK and β-tubulin and (**B**) quantitation of pERK relative to β-tubulin loading control in mutants of GNB2 (*n* = 4) and GNAQ (*n* = 2). ^*^P < 0.05, test not performed for GNAQ.

Ectopic expression of the GNAQ R183Q or Q209L in HEK293 cells is reported to cause excessive activation of the MAPK pathway (2). In line with these results, we also observed strongly enhanced ERK phosphorylation in response to GNAQ Q209L transduction of both confluent and subconfluent HUVECs, and a weaker effect of GNAQ R183Q transduction (Fig. 4). By contrast, transduction of the GNB2 K78E resulted in a statistically significant reduction in pERK levels, which was more pronounced in subconfluent cells. We also analysed the MAPKs p38 and pJNK, as well as the MAPK-activated protein kinase pS6, and observed a tendency to enhanced phosphorylation by GNAQ R183Q in HUVECs, most pronounced in subconfluent cells (Fig. 4A). Notably, these results differ from reported findings in HEK293 cells (2). By contrast, and with the exception of a weak reduction of p38 in confluent cells, we observed no modulation of these kinases in cells transduced with the GNB2 K78E mutant (Fig. 4A). Thus, the *GNB2* and *GNAQ* mutations appear to differ in their effects on MAPK signalling.

**Figure 5:**
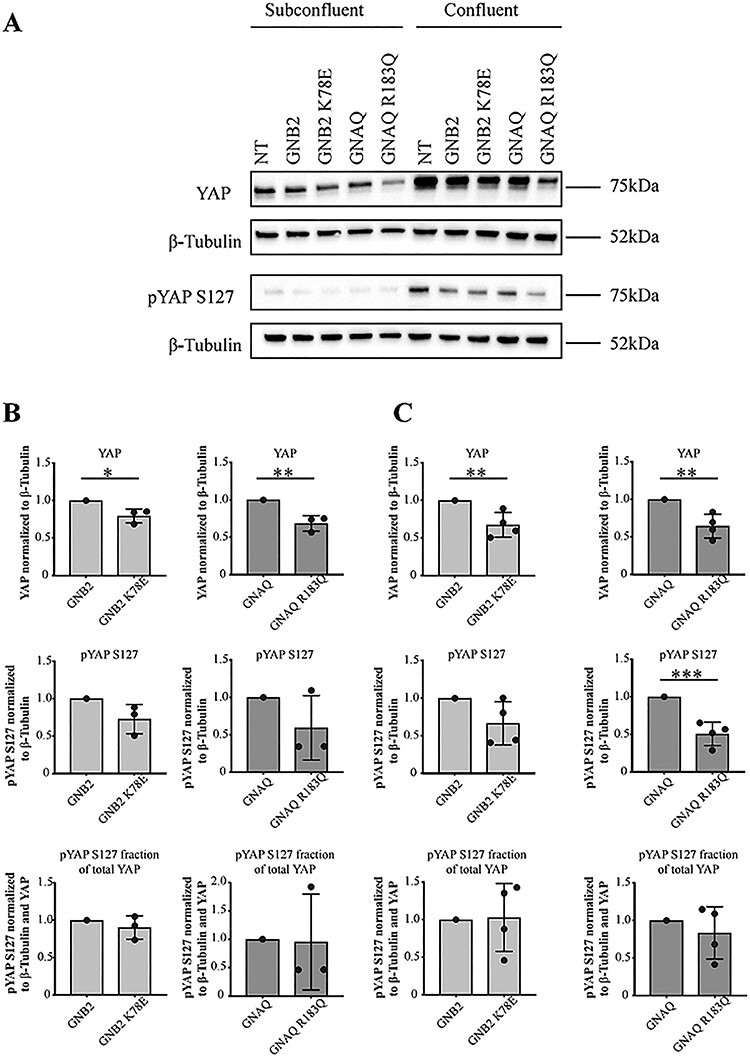
Mutations in *GNAQ* and *GNB2* reduce YAP levels. Western blot of lysates of confluent and subconfluent HUVECs transduced with adenovirus vectors as indicated by labels. (**A**) Representative blots incubated with antibodies to YAP, pYAP S127 and β-tubulin and (**B**, **C**) quantitation of YAP (top), pYAP S127 (middle) and ratio of pYAP S127 to b-tubulin over YAP to b-tubulin (bottom) in subconfluent (B, *n* = 4) and confluent cultures (C, *n* = 4). ^*^P < 0.05, ^**^P < 0.01, ^***^P < 0.001.

We next investigated the transcriptional co-activator YAP, recently found to be phosphorylated in *GNAQ* mutant cells (10,15). Western blot analyses of YAP showed reduced levels of both total YAP and YAP phosphorylated at position Ser127 (from here on referred to as pYAP S127) in GNAQ R183Q and GNB2 K78E mutants when compared to wild-type controls (Fig. 5A). Phosphorylation at Ser127 prevents YAP translocation to the nucleus, where YAP can act as a transcriptional coactivator (10). Efforts to detect nuclear translocation of YAP by means of immunocytochemistry and western blot of nuclear fractions were inconclusive (data not shown). Interestingly, the ratio of phosphorylated/total YAP remained constant, suggesting that the mutations of *GNAQ* or *GNB2* reduce expression of YAP in endothelial cells without changing the fraction of phosphorylated protein (Fig. 5B and C).

## Discussion

This study substantially expands our understanding of Sturge–Weber syndrome pathogenesis by identifying a novel somatic missense mutation in the *GNB2* gene, encoding a G-protein beta subunit (Gβ2). As the identified mutation GNB2 K78E is associated with a classical Sturge–Weber syndrome phenotype similar to the one caused by GNAQ R183Q (2), and as there is a well-characterized interaction of these G-protein subunits in GPCR-dependent intracellular signalling, it appears likely that the two mutations cause Sturge–Weber syndrome through the same downstream pathways. Intriguingly, the results of our study indicate that the MAPK pathway may be of less importance than commonly assumed and that YAP signalling, being similarly affected by the two mutations, is more relevant to the functional impairment that leads to Sturge–Weber syndrome.

Five out of the six patients in our study were positive for the GNAQ R183Q mutation, in line with the ratio of previous studies (2,4,8). In the patient negative for the known *GNAQ* mutation (patient 3), we instead identified a novel somatic mutation in the *GNB2* gene (GNB2 K78E). The variant has not previously been reported in public databases, such as the COSMIC and gnomAD databases (https://cancer.sanger.ac.uk/cosmic, https://gnomad.broadinstitute.org/), nor has the *GNB2* gene been implicated in Sturge–Weber syndrome or port-wine birthmark pathogenesis. Six additional *GNAQ* mutation-negative patients were tested for GNB2 K78E at the Kennedy Krieger Institute and Yokohama City University, but all were negative. However, strong evidence underpins the presumed pathogenicity of this mutation. First, the mutation was not observed in the unaffected biopsy of the same patient, while it was detected by both ultra-deep whole-exome and targeted amplicon sequencing in affected biopsies. Second, the phenotype of the patient was typical of Sturge–Weber syndrome, without aberrant features. Third, structural analysis (Fig. 2) and previous immunoprecipitation studies (16) indicate that GNB2 K78E interferes with the interaction between Gαq and Gβ2. Fourth, the recent discovery of a somatic missense mutation in the *GNA11* gene, encoding another G-protein alpha q subunit, corroborates the crucial role of aberrant GTPase activity in the development of vascular lesions in atypical Sturge–Weber syndrome (17,18). Finally, the *GNB2* mutation showed the same enrichment in endothelial cells as observed in *GNAQ* mutation-positive samples by us and others (3,6,19). Despite convincing evidence of pathogenicity, the fact that we have only identified the mutation in DNA from affected skin from a single patient and that we were unable to identify it in our six *GNAQ* mutation-negative controls, suggests that the mutation is a rare cause of Sturge–Weber syndrome. Replication in other patients and tissues is warranted.

The structure of heterotrimeric G-proteins and the roles of GNAQ and GNB2 within the complex explain how the two mutations may cause similar dysfunctional downstream signalling. The G-protein heterotrimer consists of the GDP-bound alpha q subunit (Gα) associated with the beta and gamma subunits (Gβϒ) (Fig. 2) and is in its resting state bound to the GPCR. Ligand-induced activation of the GPCR leads to an exchange of GDP for a GTP in the Gα subunit and subsequent dissociation from the receptor and the Gβϒ subunits. The unbound Gα subunit mediates further signalling until its GTP is hydrolysed and it reassociates with the Gβϒ subunit and the GPCR (reviewed in (20)). The primary molecular mechanism underlying Sturge–Weber syndrome was identified following the discovery of a somatic mutation in *GNAQ*, located in the GTPase pocket, inhibiting the hydrolysis of GTP and thereby causing sustained Gαq signalling (2). The novel somatic mutation GNB2 K78E provides an alternative molecular basis for increased Gαq signalling. In Figure 2, which is based on the crystal structure of the Gαβγ heterotrimer with GDP (13), the lysine at position 78 is located on the surface of the β-propeller domain of Gβ and faces the N-terminal helix of Gα where it forms an intermolecular salt bridge with the aspartic acid at position 22 (corresponding to Asp32 in Gαq). Replacement of lysine with glutamic acid will instead induce a charge repulsion, and it is therefore reasonable to assume that this mutation may disrupt the interaction between Gα and Gβϒ. Our analysis of structural data of these subunits provides strong evidence that the GNB2 K78E impairs Gαq reassociation to the Gβϒ subunit, which is likely to result in increased signalling by G-protein subunits. This is also supported by affinity precipitation-based experiments done by Yoda *et al*. (16), demonstrating that GNB2 K78E lacks the ability to bind Gαq, Gαi2, Gαi3 and Gα13.

The role of G-proteins in Sturge–Weber syndrome pathogenesis was first revealed by the discovery of GNAQ R183Q by Shirley *et al.* in 2013, who initially suggested that the mutation would lead to excessive activation of G-protein signalling by disabling the Gαq protein’s ability to autohydrolyse GTP (2). In their mechanistic considerations, Shirley *et al.* pointed to previous studies of the GNAQ Q209L in uveal melanoma, showing that the mutation caused upregulation of several MAPK pathway components, including pERK, JNK and p38 (21). The GNAQ R183Q mutation, on the other hand, resulted in only a modest increase in pERK and had no significant effect on other MAPK components (2). Later, Feng *et al.* (10) found that several activating mutations in both *GNAQ* and its paralogue *GNA11* found in uveal melanoma could also increase YAP signalling. In a review by Comi in 2016, she summarized that the current understanding of the pathogenesis of Sturge–Weber syndrome included the upregulation of both the MAPK pathway and YAP signalling, as well as mTOR indirectly (12). To identify shared downstream effects of the two Sturge–Weber syndrome mutations, we assessed the effect of GNAQ and GNB2 mutants on several MAPK pathway kinases in cultured endothelial cells, observing that GNAQ R183Q and Q209L cause a moderate to strong increase in pERK, whereas the GNB2 K78E seems to decrease pERK. We also found GNAQ R183Q and GNB2 K78E mutants to differentially affect migration as measured in scratch assays. However, both mutations found in Sturge–Weber syndrome and the *GNAQ* Q209L reduced proliferation in HUVECs. As our main hypothesis was that GNB2 K78E causes Sturge–Weber syndrome through the same mechanistic pathway as GNAQ R183Q, it is possible that altered proliferation is the underlying defect of the Sturge–Weber syndrome lesion and that ERK phosphorylation is not a key driver of altered endothelial cell behaviour involved in the Sturge–Weber syndrome phenotype. This prompted us to look for a possible involvement of the YAP pathway (10,11). Western blots of YAP showed both GNAQ R183Q and GNB2 K78E reduced levels of total YAP and pYAP S127 when compared to wild-type controls, although the ratio of phosphorylated/total protein remained constant. Nonetheless, this indicates that less nuclear YAP is available in the presence of mutated protein, which could explain the reduced proliferation mediated by both mutations. However, additional mechanisms control YAP activity in the cell, including phosphorylation (22,23), actin dynamics and mechanotransduction (24).

These aspects underline the complex regulation of YAP activity. YAP, together with TAZ (encoded by a paralogous gene of *YAP1*), are transcriptional co-activators that promote tissue growth through binding to transcriptional enhanced associated domains. They have been shown to have multiple effects on vascular cells and structures (8), in addition to regulating angiogenesis (25,26). YAP may be inhibited by the Hippo pathway through phosphorylation, in a process where GPCRs are believed to be crucial (15). However, YAP activity may also be regulated by Hippo-independent mechanisms, including mechanical stress and blood flow. Thus, whereas our findings suggest that YAP/TAZ is an essential pathway in Sturge–Weber syndrome pathogenesis, further studies are required to elaborate the specific mechanisms involved.

## Conclusions

*GNB2* is the third gene in which a somatic mutation causing Sturge–Weber syndrome has been discovered. Functional data from cultured endothelial cells indicate that the G-protein-YAP/TAZ Hippo signalling pathway is a key mechanism causing Sturge–Weber syndrome and port-wine lesions, while the MAPK pathway appears less essential. Moreover, our study confirms that GNAQ R183Q is the most common cause of Sturge–Weber syndrome and that somatic mutations in Sturge–Weber syndrome must include a localized subset of angioblasts, as the mutations were detected in all successful endothelial cultures and were enriched in laser capture microdissected blood vessels.

## Materials and Methods

### Patients

Patients were recruited from the National Centre for Rare Epilepsy-Related Disorders, where a registry of Norwegian patients with Sturge–Weber syndrome is located. Six patients with Sturge–Weber syndrome were enrolled in the study (Table 1). An additional six *GNAQ* mutation-negative patients with Sturge–Weber syndrome (five recruited from the Kennedy Krieger Institute and one from the Yokohama City University) were included for validation of novel candidate mutations. The study was approved by the Regional Ethical Committee for Medical and Health Research Ethics, South-Eastern Norway (2012/353). Informed written consent was obtained from each study participant according to the declaration of Helsinki.

### Sample collection

Two punch biopsies (4 mm) were obtained from each patient; one from unaffected skin and the other from affected skin. Biopsies were taken after administration of local anaesthesia and immediately put into Dulbecco’s modified Eagle’s medium (DMEM) supplemented with 10% fetal calf serum (FCS), 100 U/ml penicillin and 100 μg/ml streptomycin.

Biopsies from patients 1–5 were treated according to a protocol described by Normand and Karasek (27) with some modifications. Briefly, samples were swabbed sequentially with 10% iodine, 70% alcohol and Hank’s Balanced Salt Solution (HBSS) and placed with the epidermal side down in a Dispase solution (30 mg/ml in HBSS, 4°C overnight) to gently remove the epidermis and process it to establish keratinocyte cultures. A curved forceps was then used to apply pressure to the dermis approximately 10 times to push out endothelial cells and vascular stalks into a small volume of DMEM and establish endothelial cell cultures. The remaining dermis was cut into small pieces, snap-freezing one piece (approximately 1–2 mm) without further processing for DNA extraction (except for patient 1) and using the remaining pieces to establish fibroblast cultures.

Biopsies from patient 6 were bisected, processing one half in formalin/paraffin for histology and laser capture microdissection (LCM) and leaving the other half in Dispase overnight to separate dermis and epidermis before snap-freezing and subsequently extracting DNA.

### Cell cultures

#### Endothelial cultures

Medium containing vascular stalks from digested skin samples was centrifuged at 200×*g* for 5 min and the pellet resuspended in Human Endothelial-SFM (Gibco) supplemented with 0.25 mg/ml cAMP (Sigma-Aldrich, Germany), 10 ng/ml recombinant human (rh) vascular endothelial growth factor (VEGF) (R&D systems), 10 ng/ml rhEGF (R&D Systems, Minneapolis, MN) and 1 ng/ml rh fibroblast growth factor (rhFGF) (Peprotech, Europe) and seeded in a single gelatin-coated well of a 24-well plate. The next day, debris was removed by gentle washing with warm phosphate-buffered saline before adding fresh medium. Endothelial cells were obtained from four patients, but a morphologically pure endothelial culture that was allowed to passage three times before harvesting was possible in only one patient. For the remaining three patients, colonies of endothelial cells were established, but due to lack of growth these were harvested from the primary culture. In one culture, endothelial colonies failed to grow and the sample was discarded.

HUVECs were established from umbilical cords obtained from the Department of Gynaecology and Obstetrics at the Oslo University Hospital, according to a protocol approved by the Regional Committee for Research Ethics (S-05152a). Cells were isolated as described by Jaffe *et al*. (28) and cultured in MCDB 131 medium (Life Technologies, Carlsbad, CA) containing 1 ng/ml rhFGF-2, 1 ng/ml rhEGF (both from R&D Systems, Minneapolis, MN), 7.5% heat-inactivated FCS, 5 mm L-glutamine (both from Invitrogen, Waltham, MA), 1 μg/ml hydrocortisone (Sigma-Aldrich, Germany) and 50 μg/ml gentamicin, 250 ng/ml amphotericin B (both from Lonza, Basel, Switzerland), seeded on 0.1% gelatin-coated dishes, maintained at 37°C in humid 95% air/5% CO_2_ atmosphere and split at ratio 1:3. Before transduction, medium was changed to MCDB 131 containing 10 ng/ml rhFGF-2, 5 ng/ml rhEGF, 2% heat-inactivated FCS, 0.2 μg/ml hydrocortisone, 30 μg/ml gentamicin, 0.5 ng/ml rhVEGF (R&D Systems, Minneapolis, MN), 20 ng/ml insulin-like growth factor I, 1 μg/ml ascorbic acid and 0.2 μg/ml heparin (all three from Sigma-Aldrich, Germany). Confluent cells were transduced with 20 multiplicity of infection (moi) of viral vectors (Vector Biolabs, Malvern, PA, see Supplementary Material, Table S3) for 24 h and then trypsinized and replated to perform experiments. Non-transduced cells were used as controls in all experiments. Cells used in experiments were passaged up to the fourth passage.

#### Keratinocyte culture

The epidermal sheet was incubated in 1 ml of 0.05% trypsin/EDTA for 5 min, filtered through a 100 μm nylon filter and subsequently mixed with 10 ml of quenching medium (DMEM+10% FCS). The cell dispersion was spun down at 210×*g* for 5 min, resuspended in CnT-57 keratinocyte growth medium (CellnTec, Bern, Switzerland) in a single well of a 24-well plate. Cells were expanded to confluence at 75 cm^2^ after three passages.

#### Fibroblast culture

Dermal explants were placed in 6-well plates and centrifuged for 2 min at 110×*g* in order for them to attach to the well floor. Wells were then gently filled with 3 ml of DMEM +10% FCS and placed in a cell incubator. Medium was changed every 3–4 days and cells passaged 3–4 times to obtain a confluent T75 culture flask.

### Tissue preparation and LCM

Formalin-fixed paraffin-embedded skin samples were cut in 6 μm thick sections on MembraneSlide 1.0 PEN glasses (Carl Zeiss MicroImaging GmbH, Göttingen, Germany) pretreated with UV-light at 254 nm for 30 min. The sections were deparaffinized, rehydrated, haematoxylin- and eosin-stained and dehydrated according to a protocol described by Espina *et al*. (29) with the modification that tap water was used instead of Scott’s Tap Water Substitute. LCM was performed shortly after staining with a PALM Combisystem duoflex microscope system (Zeiss, Germany) equipped with Zeiss objective lenses and using PalmRobo V4.6 software. A 30 μl of Maxwell Incubation Buffer (from Maxwell 16 FFPE Plus LEV DNA Purification Kit, Promega cat. no. AS1135) was inserted in the lid of the collecting tubes (MicroTube 500, Carl Zeiss MicroImaging GmbH, Göttingen, Germany). Endothelial cells from affected tissue of patient 2 and patient 6 were marked and dissected using the laser at 200× magnification (x20 LD plan-NEOFLUAR objective lens) (Supplementary Material, Fig. S1). Controls were taken from affected epidermis of the same biopsy (patient 2) and an unaffected FFPE skin biopsy (patient 6). No more than 30 min of dissection time was used for each collecting tube, to prevent the fluid in the lid from evaporating. Cells were microdissected from two sections from patient 2 and from five sections from patient 6. Collected cells from each slide were put into separate collecting tubes that were centrifuged at 12 000×*g* at 4°C for 5 min. The affected samples from each patient were pooled, with estimated 3000–5000 cells/pooled sample. The lids of the collecting tubes were washed with additional Maxwell Incubation Buffer to achieve a total volume of 180 μl and centrifuged for 1 min. The samples were transferred to DNase/RNase free tubes and incubated at 70°C overnight in 20 μl of Proteinase K (20 μg/μl, from Maxwell kit) before mixing in 400 μl of Lysis Buffer (from Maxwell kit).

### DNA samples

DNA was extracted from LCM samples on a Maxwell AS2000, using the Maxwell 16 FFPE Plus LEV DNA Purification Kit. DNA from the remaining samples was extracted with MasterPure Complete DNA and RNA Purification Kit (Epicentre, cat. no. MC85200) according to the vendor’s protocol for cells and tissue samples. Prior to extraction, dermis samples were homogenized using a TH-02 OMNI homogenizer (OMNI International, USA) with disposable Omni-Tips (cat.no. 30750H). DNA concentrations were measured on a Qubit Fluorometer (Invitrogen) using DNA High Sensitivity Assay (Life Technologies cat. no. Q32854).

DNA from UPMM1 cell line (harbouring the *GNAQ*:c.548G>A mutation) was kindly provided by the West German Cancer Center.

### Library preparation and sequencing

Libraries for amplicon sequencing with MiSeq were prepared using a two-step PCR approach as published by Shirley *et al*. (2), resulting in targeted amplicons flanked by adapters for Illumina flow cell clustering and sequencing, and with an internal barcode introduced for each sample. Primer sequences for the amplicon covering *GNAQ*:c.548 were the same as in Shirley *et al*. (2),while primers for amplicons covering *GNB2:*c.232 and *ZNF518B*:c.614 were designed using Primer3Plus (http://primer3plus.com). All primers are listed in Supplementary Material, Table S4. DNA was amplified in a 20 μl reaction by 0.4 units Phusion High Fidelity Polymerase (New England Biolabs, cat. no. M0530S) supplemented with HF-buffer, 0.5 μm P1 primer, 0.5 μm P2 primer and 200 μm dNTPs (Applied Biosystems/ThermoFisher Scientific, cat.no. 4303442). If available, 20 ng of DNA was used as input (applying to most of the samples). For a few samples, DNA input was in the range 0.4–10 ng. Cycling conditions were as follows: 98°C for 30 s, followed by 25 cycles of 98°C for 10 s/annealing for 20 s/72°C for 10 s and finally 72°C for 5 min. Annealing temperatures were 70, 69 and 65°C for the *GNAQ*, *GNB2* and *ZNF518B* amplicons, respectively. Reactions were purified by Agencourt Ampure XP beads (Beckman Coulter, Brea, CA) according to in-house procedures, with 25 μl beads per 20 μl reaction. One-third of each purified product (or more, adjusting for DNA input) was subject to the second-step PCR using primers P3 and P4 with conditions as for first-step PCR (annealing temperature 70°C), except that number of cycles was set to 8. A few samples (mainly the LCM samples) were subject to an additional 2–4 cycles of the second PCR in order to get enough material for sequencing. Reactions were purified by AmPure XP beads. Amplicon size was evaluated on TapeStation (Agilent), using the D1000 kit (Agilent cat. no. 5067-5582/3), and concentrations were measured on Qubit. Final libraries were sequenced on Illumina MiSeq, either 50 bp single read or 150 bp paired end. The sequencing service was provided by the Norwegian Sequencing Centre (www.sequencing.uio.no).

### Bioinformatics processing of amplicon sequences

We replicated the analysis method described by Shirley *et al*. (2). In detail, the sequences from amplicon sequencing were demultiplexed according to barcode sequence and aligned against hg19 human reference genome by BWA-MEM (v0.7.12) (30). A pileup format file was generated by SAMtools (v0.1.19) (31) from the alignment BAM file. The bases at the following loci were then counted: *GNAQ* (NM_002072.3):c.548, *GNB2* (NM_005273.3):c.232 and *ZNF518B* (NM_053042.2):c.641 for corresponding samples.

### Whole-exome sequencing of the *GNAQ* mutation-negative patient

For patient 3, who was negative for the *GNAQ*-mutation, the unprocessed affected and unaffected dermis was used for whole-exome sequencing to a depth of approximately 400×. Exome capture was done using the Agilent SureSelect Human All Exon V5 kit (Agilent Technologies, Santa Clara, CA), and subsequent sequencing of the samples was done on Illumina HiSeq (Illumina, San Diego, CA), 125 bp paired end reads. The sequences were aligned to the reference human genome (hg19) by using BWA-MEM (v0.7.12) (30). The alignments were then refined by the Genome Analysis Toolkit (GATK, Version 3.4) (32–35) and PCR duplicates were marked by Picard (v1.124) (https://github.com/broadinstitute/picard). Low-quality reads were then removed from the alignments by GATK. Low-quality reads include reads without proper mate or mapping quality, or with aberrant CIGAR strings, PCR duplicates, zero or low mapping quality. The four bases were then counted on the cleaned alignment BAM file by using bam-readcount (v0.7.4) (http://github.com/genome/bam-readcount). A variant was called wherever three or more reads showed the same non-reference allele. The variant file was annotated by Annovar (v2015Jun17) (35).

### Variant filtration

The annotated variants were analysed using the software FILTUS (36) (http://folk.uio.no/magnusv/filtus.html). Variants from affected tissue were filtered on the following criteria: alternative read count at least 15, allele frequency below 1% in the 1000 Genomes Project database (http://www.1000genomes.org) and GERP score above 5. The remaining variants were then filtered against the variants from unaffected tissue with an alternative read count over 5. This yielded a candidate list of 55 variants, which were inspected manually in IGV (https://www.broadinstitute.org/igv/). Most variants resulted from reads of poor quality and had low base calling quality. Only two variants were found to be non-artefacts, stemming from long reads of high quality with good single base calling quality.

### Structural analysis of the GNB2 protein

Structural analysis of the GNB2 protein was based on the 2.0 Å crystal structure of a heterotrimeric G-protein (PDB ID: 1GOT) (13), and Figure 2 made with PYMOL (http://www.pymol.org).

### Cell proliferation assay

Transduced HUVECs were seeded in 96-well plates with detachable strips of eight wells at 19 × 10^3^ cells/cm^2^ in three replicate wells, fixing cells daily in 100 μl cold 4% paraformaldehyde (Chemi-Teknik, Oslo, Norway) at room temperature, 10 min. Cells were subsequently dried for 20 min and stored at 4°C until the end of the experiment. Fixed cells were next stained with 70 μl freshly made 0.1% crystal violet (Apotekforeningen, Oslo, Norway) in distilled water (4 min, RT), before extensively washing them under cold tap water. Nuclear dye was eluted with 100 μl 33% acetic acid (Sigma-Aldrich, Germany) and absorbance was read at 550 nm in an Epoch microplate reader with software Gen5.0.10.1 (BioTek, Winooski, VT).

### Cell migration assay

Transduced HUVECs were seeded to 24-well plates at 50 × 10^3^ cells/cm^2^ in at least three wells per experimental condition. The cell migration assay was performed at confluence by scraping a straight line with a sterile 200 μl pipet tip in the cell monolayer. Detached cells and debris were replaced with fresh medium before incubation at 37°C for 6 h. Images from one region of the scraped area in each well were taken under a fluorescence microscope (Evos® FL Cell Imagine System, Thermo Fisher Scientific, Waltham, MA) at 10× magnification at 0, 2, 4 and 6 h (Supplementary Material, Fig. S2). To determine the rate of cell migration, images were analysed using ImageJ 1.50i software (National Institutes of Health, Bethesda, MD) (ImageJ, NIH), subtracting the free area after migration from the area after scraping, according to Buachan *et al*. (37).

### Immunoblotting

Transduced subconfluent or confluent HUVECs were lysed on ice with cold lysis buffer (62.5 mm Tris–HCl, 6.8 pH, 5% SDS, 10% glycerol) containing 1:100 (*v/v*) freshly added Halt Protease & Phosphatase Inhibitor Single-Use Cocktail, EDTA-free (Thermo Fisher Scientific, Waltham, MA) and homogenized using QIAshredder columns (QIAGEN, Valencia, CA). Protein concentrations were measured using Pierce™ BCA Protein Assay Kit (Thermo Fisher Scientific, Waltham, MA) at 556 nm in Epoch microplate reader with software Gen5.0.10.1 (BioTek). Sample buffer (0.01% BPB, 5% 2ME) was added to the cell lysates. After incubation for 10 min at 75°C, 10 μg of protein was loaded on MiniPROTEAN®TGX™ Precast Gels (4–20%), run in electrophoresis buffer (248 mm Tris, 14.4% glycine, 1% SDS) at 200 V, 30 min in Bio-Rad System and transferred to 0.2 μm nitrocellulose membranes using the Trans-Blot® Turbo™ Transfer System (gels and membranes from Bio-Rad Laboratories, Hercules, CA). Membranes were blocked with 5% Blotting-Grade Blocker (Bio-Rad Laboratories, Hercules, CA) or with 5% BSA (Sigma-Aldrich, Germany) when using antibodies against phosphorylated protein. Membranes were incubated on a shaker with primary antibodies overnight at 4°C and with secondary antibodies 2 h at room temperature (antibodies listed in Supplementary Material, Table S5), all diluted with 1% BSA (Sigma-Aldrich, Germany) or 1% Blotting-Grade Blocker (Bio-Rad Laboratories, Hercules, CA) in TBST (pH 7.4, 10 mm Tris, 137 mm NaCl, 1 M HCl, 0.1% Tween20). Proteins were detected using SuperSignal West Dura Extended Duration Substrate (Thermo Fisher Scientific, Waltham, MA) or Luminata™ Forte Western HRP Substrate (Millipore Corporation, Billerica, MA). Full Range Rainbow™ Recombinant Protein Molecular Weight Marker (GE Healthcare Life Science) was detected using Magic Pen Glow in the Dark Phosphorescent (Marvy Uchida, Torrance, CA). Proteins were visualized using the ChemiDoc MP System (Bio-Rad Laboratories, Hercules, CA). Membranes were stripped with Restore™ PLUS Western Blot Stripping Buffer (Thermo Fisher Scientific, Waltham, MA). Images were processed by software Image Lab Version 4.1 build 16 (Bio-Rad Laboratories, Hercules, CA) for densitometric quantitation. All the values were normalized to β-tubulin.

### Statistical analysis

In the functional experiments, statistical comparisons were performed with the two-tailed Student’s *t*-test after passing tests for normal distribution using GraphPad Prism 8.0.2 software. Dispersion of results was expressed as mean ± SEM or mean ± SD as appropriate and indicated in figure legends.

## Supplementary Material

HMG-2021-D-00032_Fjaer_SupplementaryMaterial_ddab144

## References

[1] Happle, R. (1987) Lethal genes surviving by mosaicism: a possible explanation for sporadic birth defects involving the skin. J. Am. Acad. Dermatol., 16, 899–906. 3033033 10.1016/s0190-9622(87)80249-9

[2] Shirley, M.D., Tang, H., Gallione, C.J., Baugher, J.D., Frelin, L.P., Cohen, B., North, P.E., Marchuk, D.A., Comi, A.M. and Pevsner, J. (2013) Sturge-Weber syndrome and port-wine stains caused by somatic mutation in *GNAQ*. N. Engl. J. Med., 368, 1971–1979. 23656586 10.1056/NEJMoa1213507 PMC3749068

[3] Couto, J.A., Huang, L., Vivero, M.P., Kamitaki, N., Maclellan, R.A., Mulliken, J.B., Bischoff, J., Warman, M.L. and Greene, A.K. (2016) Endothelial cells from capillary malformations are enriched for somatic *GNAQ* mutations. Plast. Reconstr. Surg., 137, 77e–82e. 10.1097/PRS.0000000000001868 PMC5242181 26368330

[4] Frigerio, A., Wright, K., Wooderchak-Donahue, W., Tan, O.T., Margraf, R., Stevenson, D.A., Grimmer, J.F. and Bayrak-Toydemir, P. (2015) Genetic variants associated with port-wine stains. PLoS One, 10, e0133158. 26192947 10.1371/journal.pone.0133158 PMC4508108

[5] Hildebrand, M.S., Harvey, A.S., Malone, S., Damiano, J.A., Do, H., Ye, Z., McQuillan, L., Maixner, W., Kalnins, R., Nolan, B. et al. (2018) Somatic *GNAQ* mutation in the forme fruste of Sturge-Weber syndrome. Neurol. Genet., 4, e236. 29725622 10.1212/NXG.0000000000000236 PMC5931068

[6] Huang, L., Couto, J.A., Pinto, A., Alexandrescu, S., Madsen, J.R., Greene, A.K., Sahin, M. and Bischoff, J. (2017) Somatic *GNAQ* mutation is enriched in brain endothelial cells in Sturge-weber syndrome. Pediatr. Neurol., 67, 59–63. 27919468 10.1016/j.pediatrneurol.2016.10.010 PMC5303551

[7] Lian, C.G., Sholl, L.M., Zakka, L.R., Teresa, O.M., Liu, C., Xu, S., Stanek, E., Garcia, E., Jia, Y., MacConaill, L.E. et al. (2014) Novel genetic mutations in a sporadic port-wine stain. JAMA Dermatol., 150, 1336–1340. 25188413 10.1001/jamadermatol.2014.1244

[8] Nakashima, M., Miyajima, M., Sugano, H., Iimura, Y., Kato, M., Tsurusaki, Y., Miyake, N., Saitsu, H., Arai, H. and Matsumoto, N. (2014) The somatic *GNAQ* mutation c.548G>a (p.R183Q) is consistently found in Sturge-Weber syndrome. J. Hum. Genet., 59, 691–693. 25374402 10.1038/jhg.2014.95

[9] Comi, A.M. (2003) Pathophysiology of Sturge-Weber syndrome. J. Child Neurol., 18, 509–516. 13677575 10.1177/08830738030180080701

[10] Feng, X., Degese, M.S., Iglesias-Bartolome, R., Vaque, J.P., Molinolo, A.A., Rodrigues, M., Zaidi, M.R., Ksander, B.R., Merlino, G., Sodhi, A. et al. (2014) Hippo-independent activation of YAP by the *GNAQ* uveal melanoma oncogene through a trio-regulated rho GTPase signaling circuitry. Cancer Cell, 25, 831–845. 24882515 10.1016/j.ccr.2014.04.016 PMC4074519

[11] Yu, F.X., Luo, J., Mo, J.S., Liu, G., Kim, Y.C., Meng, Z., Zhao, L., Peyman, G., Ouyang, H., Jiang, W. et al. (2014) Mutant Gq/11 promote uveal melanoma tumorigenesis by activating YAP. Cancer Cell, 25, 822–830. 24882516 10.1016/j.ccr.2014.04.017 PMC4075337

[12] Comi, A.M., Sahin, M., Hammill, A., Kaplan, E.H., Juhász, C., North, P., Ball, K.L., Levin, A.V., Cohen, B., Morris, J. et al. (2016) Leveraging a Sturge-Weber gene discovery: an agenda for future research. Pediatr. Neurol., 58, 12–24. 27268758 10.1016/j.pediatrneurol.2015.11.009 PMC5509161

[13] Lambright, D.G., Sondek, J., Bohm, A., Skiba, N.P., Hamm, H.E. and Sigler, P.B. (1996) The 2.0 a crystal structure of a heterotrimeric G protein. Nature, 379, 311–319. 8552184 10.1038/379311a0

[14] Comati, A., Beck, H., Halliday, W., Snipes, G.J., Plate, K.H. and Acker, T. (2007) Upregulation of hypoxia-inducible factor (HIF)-1alpha and HIF-2alpha in leptomeningeal vascular malformations of Sturge-weber syndrome. J. Neuropathol. Exp. Neurol., 66, 86–97. 17204940 10.1097/nen.0b013e31802d9011

[15] Yu, F.X., Zhao, B., Panupinthu, N., Jewell, J.L., Lian, I., Wang, L.H., Zhao, J., Yuan, H., Tumaneng, K., Li, H. et al. (2012) Regulation of the hippo-YAP pathway by G-protein-coupled receptor signaling. Cell, 150, 780–791. 22863277 10.1016/j.cell.2012.06.037 PMC3433174

[16] Yoda, A., Adelmant, G., Tamburini, J., Chapuy, B., Shindoh, N., Yoda, Y., Weigert, O., Kopp, N., Wu, S.C., Kim, S.S. et al. (2015) Mutations in G protein β subunits promote transformation and kinase inhibitor resistance. Nat. Med., 21, 71–75. 25485910 10.1038/nm.3751 PMC4289115

[17] Polubothu, S., Al-Olabi, L., Carmen Del Boente, M., Chacko, A., Eleftheriou, G., Glover, M., Jiménez-Gallo, D., Jones, E.A., Lomas, D., Fölster-Holst, R. et al. (2020) *GNA11* mutation as a cause of Sturge-Weber syndrome: expansion of the phenotypic Spectrum of G(α/11) mosaicism and the associated clinical diagnoses. J. Invest. Dermatol., 140, 1110–1113. 31838126 10.1016/j.jid.2019.10.019 PMC7187890

[18] Thorpe, J., Frelin, L.P., McCann, M., Pardo, C.A., Cohen, B.A., Comi, A.M. and Pevsner, J. (2021) Identification of a mosaic activating mutation in *GNA11* in atypical Sturge-Weber syndrome. J. Invest. Dermatol., 141, 685–688. 32771470 10.1016/j.jid.2020.03.978 PMC8483769

[19] Sundaram, S.K., Michelhaugh, S.K., Klinger, N.V., Kupsky, W.J., Sood, S., Chugani, H.T., Mittal, S. and Juhász, C. (2017) GNAQ mutation in the venous vascular malformation and underlying brain tissue in Sturge-Weber syndrome. Neuropediatrics, 48, 385–389. 28571101 10.1055/s-0037-1603515 PMC5587372

[20] Oldham, W.M. and Hamm, H.E. (2008) Heterotrimeric G protein activation by G-protein-coupled receptors. Nat. Rev. Mol. Cell Biol., 9, 60–71. 18043707 10.1038/nrm2299

[21] Van Raamsdonk, C.D., Bezrookove, V., Green, G., Bauer, J., Gaugler, L., O'Brien, J.M., Simpson, E.M., Barsh, G.S. and Bastian, B.C. (2009) Frequent somatic mutations of GNAQ in uveal melanoma and blue naevi. Nature, 457, 599–602. 19078957 10.1038/nature07586 PMC2696133

[22] Varelas, X. (2014) The hippo pathway effectors TAZ and YAP in development, homeostasis and disease. Development, 141, 1614–1626. 24715453 10.1242/dev.102376

[23] Wang, Y., Justilien, V., Brennan, K.I., Jamieson, L., Murray, N.R. and Fields, A.P. (2017) PKCι regulates nuclear YAP1 localization and ovarian cancer tumorigenesis. Oncogene, 36, 534–545. 27321186 10.1038/onc.2016.224 PMC5173453

[24] Gumbiner, B.M. and Kim, N.G. (2014) The hippo-YAP signaling pathway and contact inhibition of growth. J. Cell Sci., 127, 709–717. 24532814 10.1242/jcs.140103 PMC3924201

[25] Azad, T., Ghahremani, M. and Yang, X. (2019) The role of YAP and TAZ in angiogenesis and vascular mimicry. Cells, 8, 407. 10.3390/cells8050407 PMC6562567 31052445

[26] Boopathy, G.T.K. and Hong, W. (2019) Role of hippo pathway-YAP/TAZ signaling in angiogenesis. Front. Cell Dev. Biol., 7, 49. 31024911 10.3389/fcell.2019.00049 PMC6468149

[27] Normand, J. and Karasek, M.A. (1995) A method for the isolation and serial propagation of keratinocytes, endothelial cells, and fibroblasts from a single punch biopsy of human skin. In Vitro Cell Dev. Biol. Anim., 31, 447–455. 8589888 10.1007/BF02634257

[28] Jaffe, E.A., Nachman, R.L., Becker, C.G. and Minick, C.R. (1973) Culture of human endothelial cells derived from umbilical veins. Identification by morphologic and immunologic criteria. J. Clin. Invest., 52, 2745–2756. 4355998 10.1172/JCI107470 PMC302542

[29] Espina, V., Wulfkuhle, J.D., Calvert, V.S., VanMeter, A., Zhou, W., Coukos, G., Geho, D.H., Petricoin, E.F., 3rd and Liotta, L.A. (2006) Laser-capture microdissection. Nat. Protoc., 1, 586–603. 17406286 10.1038/nprot.2006.85

[30] Li, H. (2013) Aligning sequence reads, clone sequences and assembly contigs with BWA-MEM. arXiv preprint, arXiv:1303.3997.

[31] Li, H., Handsaker, B., Wysoker, A., Fennell, T., Ruan, J., Homer, N., Marth, G., Abecasis, G. and Durbin, R. (2009) The Sequence Alignment/Map format and SAMtools. Bioinformatics, 25, 2078–2079. 19505943 10.1093/bioinformatics/btp352 PMC2723002

[32] DePristo, M.A., Banks, E., Poplin, R., Garimella, K.V., Maguire, J.R., Hartl, C., Philippakis, A.A., del Angel, G., Rivas, M.A., Hanna, M. et al. (2011) A framework for variation discovery and genotyping using next-generation DNA sequencing data. Nat. Genet., 43, 491–498. 21478889 10.1038/ng.806 PMC3083463

[33] McKenna, A., Hanna, M., Banks, E., Sivachenko, A., Cibulskis, K., Kernytsky, A., Garimella, K., Altshuler, D., Gabriel, S., Daly, M. et al. (2010) The Genome Analysis Toolkit: a MapReduce framework for analyzing next-generation DNA sequencing data. Genome Res., 20, 1297–1303. 20644199 10.1101/gr.107524.110 PMC2928508

[34] Van der Auwera, G.A., Carneiro, M.O., Hartl, C., Poplin, R., Del Angel, G., Levy-Moonshine, A., Jordan, T., Shakir, K., Roazen, D., Thibault, J. *et al.* (2013) From FastQ data to high confidence variant calls: the Genome Analysis Toolkit best practices pipeline. Curr. Protoc. Bioinformatics, 43, 11.10.11-11.10.33. 25431634 10.1002/0471250953.bi1110s43 PMC4243306

[35] Wang, K., Li, M. and Hakonarson, H. (2010) ANNOVAR: functional annotation of genetic variants from high-throughput sequencing data. Nucleic Acids Res., 38, e164. 20601685 10.1093/nar/gkq603 PMC2938201

[36] Vigeland, M.D., Gjøtterud, K.S. and Selmer, K.K. (2016) FILTUS: a desktop GUI for fast and efficient detection of disease-causing variants, including a novel autozygosity detector. Bioinformatics, 32, 1592–1594. 26819469 10.1093/bioinformatics/btw046 PMC4866527

[37] Buachan, P., Chularojmontri, L. and Wattanapitayakul, S. (2014) Selected activities of *Citrus maxima* Merr. fruits on human endothelial cells: enhancing cell migration and delaying cellular aging. Nutrients, 6, 1618–1634. 24763109 10.3390/nu6041618 PMC4011055

