# Supplementary material for: A novel somatic mutation in GNB2 provides new insights to the pathogenesis of Sturge–Weber syndrome
Source: Hum Mol Genet. 2021 Jun 14;30(21):1919–31. doi: 10.1093/hmg/ddab144 (PMC8522634; doi:10.1093/hmg/ddab144)
Supplement: HMG-2021-D-00032_Fjaer_SupplementaryMaterial_ddab144 [file hmg-2021-d-00032_fjaer_supplementarymaterial_ddab144.pdf]

Supplementary material of:

A novel somatic mutation in *GNB2* provides new insights to the pathogenesis of Sturge-Weber syndrome

Roar Fjær, Katarzyna Marciniak,* Olav Sundnes,* Hanne Hjorthaug, Ying Sheng, Clara Hammarström, Jan Cezary Sitek, Magnus Dehli Vigeland, Paul Hoff Backe, Ane-Marte Øye, Johanna Hol, Tor Espen Bendvold, Yuri Uchiyama, Naomichi Matsumoto, Anne Comi, Jonathan Pevsner, Guttorm Haraldsen,[†] and Kaja Kristine Selmer[1,2,3,†]

[† and *] These authors contributed equally to this work.

1. Department of Medical Genetics, Oslo University Hospital and University of Oslo, Oslo,
2. National Centre for Rare Epilepsy-Related Disorders, Oslo University Hospital and the University of Oslo, Oslo, Norway
3. Department of Research and Innovation, Division of Clinical Neuroscience, Oslo University Hospital, Oslo, Norway

Correspondence to:
Kaja Selmer, MD, PhD, e-mail: k.k.selmer@medisin.uio.no.

Supplementary figure 1.

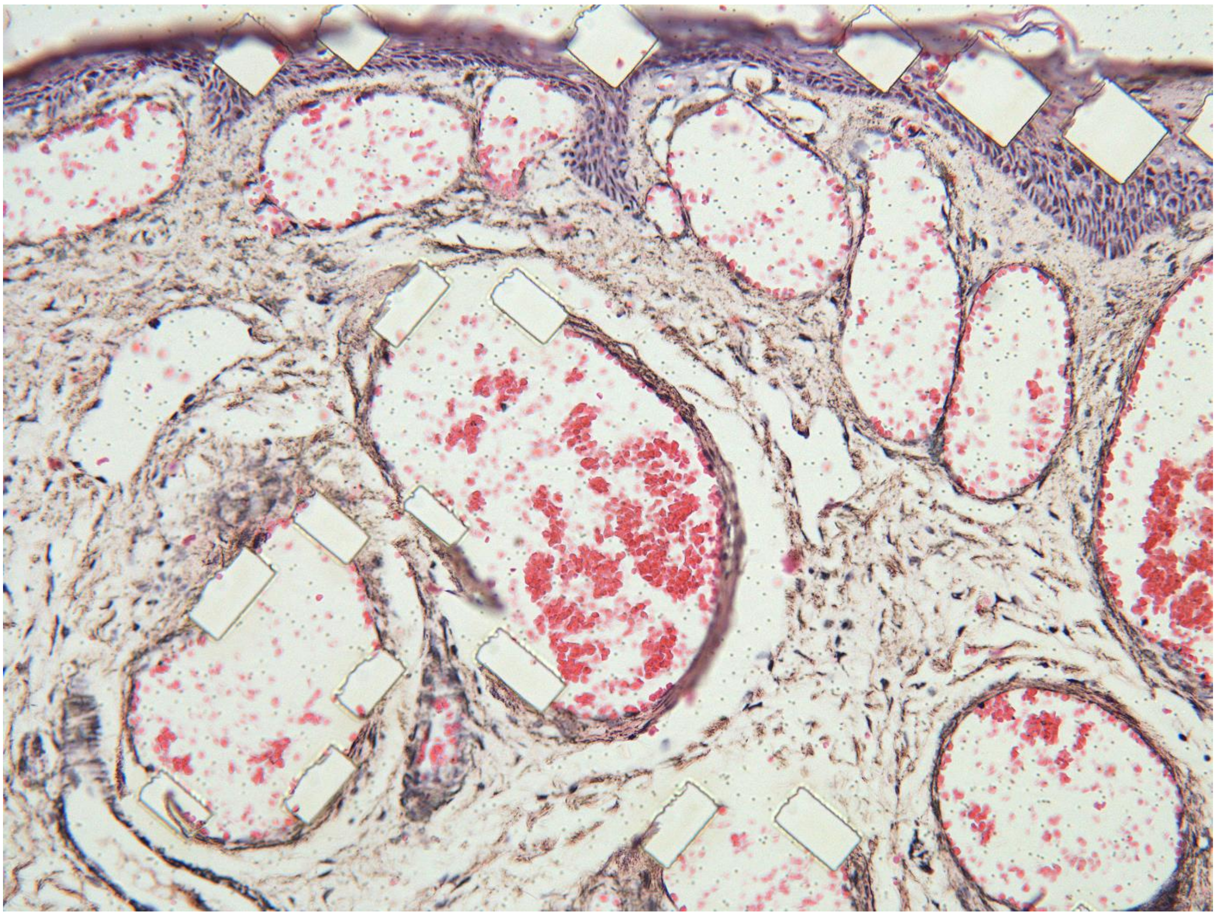

Supplementary Figure 1. Light microscopy image of laser microdissection sample. Sample from patient 2, demonstrating the dissected samples of endothelial cells and epithelial cells as controls.

Supplementary Figure 2.

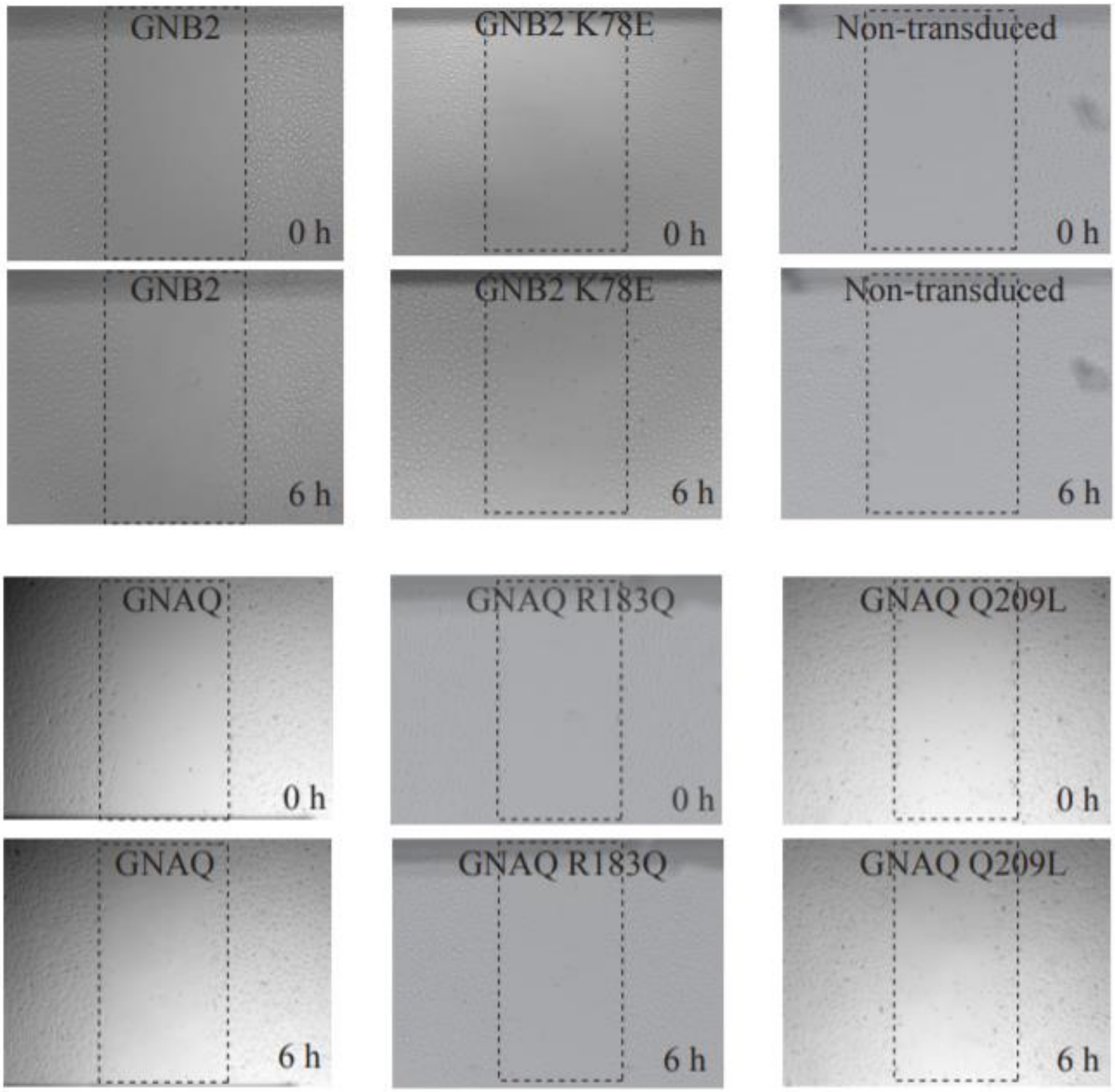

Supplementary Figure 2. Representative pictures of cell migration scratch assays. Pictures at 0h and 6h.

Supplementary Table 1. Results from MiSeq sequencing of GNAQ:c.548G>A, all samples.

Patient	Sample	Mutation frequency	Read depth	G (ref)	A (mut)	T	C
**1**	Affected endothelial culture	14.36	130299	111585	18709	4	1
	Affected keratinocyte culture	0.01	144114	144091	16	4	3
	Affected fibroblast culture	0.02	120704	120665	24	13	2
	Unaffected keratinocyte culture	0.01	102340	102323	7	10	0
	Blood sample	0.01	145856	145829	13	10	4
**2**	Affected dermis	7.08	128341	119233	9091	16	1
	Affected endothelial culture	9.28	100355	91036	9314	5	0
	Affected keratinocyte culture	0.01	145606	145579	15	10	2
	Affected fibroblast culture	0.01	133385	133364	17	3	1
	LCM affected endothelium	29.88	60684	42552	18131	1	0
	LCM affected epithelium	0.01	41671	41664	5	2	0
	Unaffected dermis	0.02	125925	125889	25	10	1
	Unaffected keratinocyte culture	0.01	114986	114973	8	5	0
	Unaffected fibroblast culture	0.01	105876	105852	13	10	1
**3**	Affected endothelial culture	0.01	99182	99159	14	6	3
	Affected keratinocyte culture	0.01	147322	147297	14	11	0
	Affected fibroblast culture	0.01	130421	130399	11	7	4
	Affected dermis	0.01	131325	131300	16	8	1
	Unaffected keratinocyte culture	0.02	128006	127973	22	6	5
	Unaffected fibroblast culture	0.01	116201	116184	8	8	1
	Unaffected dermis	0.01	111944	111919	16	9	0
**4**	Affected dermis	7.39	135365	125357	9998	8	2
	Affected endothelial culture 1	0.24	113991	113714	269	5	3
	Affected endothelial culture 2	0.13	143019	142825	189	4	1
	Affected keratinocyte culture	0.02	150899	150857	30	11	1
	Affected fibroblast culture	0.01	132793	132762	19	11	1
	Unaffected dermis	0.03	97605	97567	34	1	3
	Unaffected endothelial culture	0.01	103627	103608	13	6	0
	Unaffected keratinocyte culture	0.05	108155	108087	56	10	2
	Unaffected fibroblast culture	0.01	84837	84818	10	8	1
**5**	Affected dermis	8.98	147823	134533	13276	12	2
	Affected keratinocyte culture	0.01	130270	130245	17	5	3
	Affected fibroblast culture	0.01	120055	120036	14	5	0
	Unaffected dermis	1.06	89863	88907	949	5	2
	Unaffected keratinocyte culture	0.02	117723	117686	28	7	2
	Unaffected fibroblast culture	0.01	110664	110643	12	8	1
**6**	Affected dermis	5.72	99490	93794	5694	0	2
	LCM affected endothelium	19.93	192942	154496	38444	0	2
	Affected epithelium	2.70	107744	104832	2907	4	1
	Unaffected dermis FFPE	0.01	163311	163283	21	4	3
	Unaffected dermis	0.21	88327	88142	183	1	1
	Unaffected epithelium	1.08	83844	82933	907	3	1
**Positive control**	UPMM1 cells GNAQ c.548G>A	99.98	87850	16	87830	3	1

Supplementary Table 2. P-values for the proliferation and migration analyses in Fig 3.

Analysis	Comparison	P value
**Proliferation (Fig. 2A and B)**	GNB2 WT vs. GNB2 K78E	.1781
	GNAQ WT vs. GNAQ R183Q	.0043
	GNAQ WT vs. GNAQ Q209L	.0054
**Migration (Fig. 2C)**	GNB2 WT vs. GNB2 K78E	.0384
	GNAQ WT vs. GNAQ R183Q	.0227
	GNAQ WT vs. GNAQ Q209L	.1543

WT=wild-type; vs.=versus.

Supplementary Table 3. Name and composition of viral vectors

Name of viral vector construct	Composition of viral vector construct
Ad5-GFP-hGNAQ-wt	Adenovirus type 5 (dE1/E3) encoding green fluorescent protein (GFP) and human wt GNAQ under separate CMV promoters
Ad5-mCherry-hGNAQ-R183Q	Adenovirus type 5 encoding under separate CMV promoters red fluorescent protein (mCherry) and with sequence of human GNAQ gen mutated to change arginine to glutamine at position 183
Ad5-mCherry-hGNAQ-Q209L	Adenovirus type 5 (dE1/E3) encoding under separate CMV promoters red fluorescentprotein (mCherry) and human GNAQ gene mutated to change glutamine to leucine at position 209
Ad5-GFP-hGNB2-wt	Adenovirus type 5 (dE1/E3) encoding green fluorescent protein (GFP) and human wt GNB2 under separate CMV promoters
Ad5-GFP-hGNB2-K78E	Adenovirus type 5 (dE1/E3) encoding under separate CMV promoters green fluorescent protein (GFP) and human GNB2 gene with mutation changing Lysine to Glutamic acid in protein chain at position 78

Supplementary Table 4. Primers for MiSeq library preparation.

Primer name	Sequence (5' to 3')
P4	CAAGCAGAAGACGGCATACGAGATCGGTCTCGGCATTCCTGCTGAACCGCTCTTCCGATCT
P3	AATGATACGGCGACCACCGAGATCTACACTCTTTCCCTACACGACGCTCTTCCGATCT
GNAQ_P2	CTCGGCATTCCTGCTGAACCGCTCTTCCGATCTCCTTTCCGTAGACAGCTTTGGTGTGATG
GNAQ_P1_1	ACACTCTTTCCCTACACGACGCTCTTCCGATCTGATCTTATAACGGGTATTCGATGATCCCTGTGGTGGG
GNAQ_P1_2	ACACTCTTTCCCTACACGACGCTCTTCCGATCTATCGTTAATTGGGGTATTCGATGATCCCTGTGGTGGG
GNAQ_P1_3	ACACTCTTTCCCTACACGACGCTCTTCCGATCTTCGAAAGTTTTGGGTATTCGATGATCCCTGTGGTGGG
GNAQ_P1_4	ACACTCTTTCCCTACACGACGCTCTTCCGATCTCGATTATTATGGGGTATTCGATGATCCCTGTGGTGGG
GNAQ_P1_5	ACACTCTTTCCCTACACGACGCTCTTCCGATCTGATCATTAGTTGGGTATTCGATGATCCCTGTGGTGGG
GNAQ_P1_6	ACACTCTTTCCCTACACGACGCTCTTCCGATCTATCGATTTTAGGGGTATTCGATGATCCCTGTGGTGGG
GNAQ_P1_7	ACACTCTTTCCCTACACGACGCTCTTCCGATCTTCGATATATGTGGGTATTCGATGATCCCTGTGGTGGG
GNAQ_P1_8	ACACTCTTTCCCTACACGACGCTCTTCCGATCTCGATCTATAGTGGGTATTCGATGATCCCTGTGGTGGG
GNAQ_P1_9	ACACTCTTTCCCTACACGACGCTCTTCCGATCTGATCAAATCCTGGGTATTCGATGATCCCTGTGGTGGG
GNAQ_P1_10	ACACTCTTTCCCTACACGACGCTCTTCCGATCTATCGTCAACTAGGGTATTCGATGATCCCTGTGGTGGG
GNAQ_P1_11	ACACTCTTTCCCTACACGACGCTCTTCCGATCTTCGATCATGATGGGTATTCGATGATCCCTGTGGTGGG
GNAQ_P1_12	ACACTCTTTCCCTACACGACGCTCTTCCGATCTCGATCTAATCAGGGTATTCGATGATCCCTGTGGTGGG
GNAQ_P1_13	ACACTCTTTCCCTACACGACGCTCTTCCGATCTGATCAAACTTCGGGTATTCGATGATCCCTGTGGTGGG
GNAQ_P1_14	ACACTCTTTCCCTACACGACGCTCTTCCGATCTATCGGGATTTTGGGTATTCGATGATCCCTGTGGTGGG
GNAQ_P1_15	ACACTCTTTCCCTACACGACGCTCTTCCGATCTTCGAATCAACTGGGTATTCGATGATCCCTGTGGTGGG
GNAQ_P1_16	ACACTCTTTCCCTACACGACGCTCTTCCGATCTCGATTACCATAGGGTATTCGATGATCCCTGTGGTGGG
GNAQ_P1_17	ACACTCTTTCCCTACACGACGCTCTTCCGATCTGATCTACACATGGGTATTCGATGATCCCTGTGGTGGG
GNAQ_P1_18	ACACTCTTTCCCTACACGACGCTCTTCCGATCTATCGATCCTAAGGGTATTCGATGATCCCTGTGGTGGG
GNAQ_P1_19	ACACTCTTTCCCTACACGACGCTCTTCCGATCTTCGATTGCAATGGGTATTCGATGATCCCTGTGGTGGG
GNAQ_P1_20	ACACTCTTTCCCTACACGACGCTCTTCCGATCTCGATTTGGTTAGGGTATTCGATGATCCCTGTGGTGGG
GNAQ_P1_21	ACACTCTTTCCCTACACGACGCTCTTCCGATCTGATCCATTACAGGGTATTCGATGATCCCTGTGGTGGG
GNAQ_P1_22	ACACTCTTTCCCTACACGACGCTCTTCCGATCTATCGACTAATCGGGTATTCGATGATCCCTGTGGTGGG
GNAQ_P1_23	ACACTCTTTCCCTACACGACGCTCTTCCGATCTTCGAGTTGATTGGGTATTCGATGATCCCTGTGGTGGG
GNAQ_P1_24	ACACTCTTTCCCTACACGACGCTCTTCCGATCTCGATACTTCAAGGGTATTCGATGATCCCTGTGGTGGG
GNAQ_P1_25	ACACTCTTTCCCTACACGACGCTCTTCCGATCTGATCTGTTGTAGGGTATTCGATGATCCCTGTGGTGGG
GNAQ_P1_26	ACACTCTTTCCCTACACGACGCTCTTCCGATCTATCGCATATACGGGTATTCGATGATCCCTGTGGTGGG
GNAQ_P1_27	ACACTCTTTCCCTACACGACGCTCTTCCGATCTTCGATGTGTATGGGTATTCGATGATCCCTGTGGTGGG
GNAQ_P1_28	ACACTCTTTCCCTACACGACGCTCTTCCGATCTCGATGTTTTGAGGGTATTCGATGATCCCTGTGGTGGG
GNAQ_P1_29	ACACTCTTTCCCTACACGACGCTCTTCCGATCTGATCCCACAATGGGTATTCGATGATCCCTGTGGTGGG
GNAQ_P1_30	ACACTCTTTCCCTACACGACGCTCTTCCGATCTATCGTTAGCCAGGGTATTCGATGATCCCTGTGGTGGG
GNAQ_P1_31	ACACTCTTTCCCTACACGACGCTCTTCCGATCTTCGAGAATCTCGGGTATTCGATGATCCCTGTGGTGGG
GNAQ_P1_32	ACACTCTTTCCCTACACGACGCTCTTCCGATCTCGATAGACCTTGGGTATTCGATGATCCCTGTGGTGGG
GNAQ_P1_33	ACACTCTTTCCCTACACGACGCTCTTCCGATCTGATCTTACGGTGGGTATTCGATGATCCCTGTGGTGGG
GNAQ_P1_34	ACACTCTTTCCCTACACGACGCTCTTCCGATCTATCGAGATTCCGGGTATTCGATGATCCCTGTGGTGGG
GNAQ_P1_35	ACACTCTTTCCCTACACGACGCTCTTCCGATCTTCGAGAACTCTGGGTATTCGATGATCCCTGTGGTGGG
GNAQ_P1_36	ACACTCTTTCCCTACACGACGCTCTTCCGATCTCGATCCAGTTAGGGTATTCGATGATCCCTGTGGTGGG
GNAQ_P1_37	ACACTCTTTCCCTACACGACGCTCTTCCGATCTGATCTGCTACAGGGTATTCGATGATCCCTGTGGTGGG
GNAQ_P1_38	ACACTCTTTCCCTACACGACGCTCTTCCGATCTATCGGTCAATCGGGTATTCGATGATCCCTGTGGTGGG
GNAQ_P1_39	ACACTCTTTCCCTACACGACGCTCTTCCGATCTTCGAACCGATTGGGTATTCGATGATCCCTGTGGTGGG
GNAQ_P1_40	ACACTCTTTCCCTACACGACGCTCTTCCGATCTCGATGTCTCAAGGGTATTCGATGATCCCTGTGGTGGG
GNAQ_P1_41	ACACTCTTTCCCTACACGACGCTCTTCCGATCTGATCCACTGTAGGGTATTCGATGATCCCTGTGGTGGG
GNB2_P2	CTCGGCATTCCTGCTGAACCGCTCTTCCGATCTCGGGGTCAGAGCCAGCCAG
GNB2 P1_46	ACACTCTTTCCCTACACGACGCTCTTCCGATCTATCGCTGACTATGCACAACAGAGAGAGACCCTACCTT
GNB2 P1_47	ACACTCTTTCCCTACACGACGCTCTTCCGATCTTCGACTGTGATTGCACAACAGAGAGAGACCCTACCTT
GNB2 P1_48	ACACTCTTTCCCTACACGACGCTCTTCCGATCTCGATTCGATCATGCACAACAGAGAGAGACCCTACCTT
GNB2 P1_49	ACACTCTTTCCCTACACGACGCTCTTCCGATCTGATCATTGACCTGCACAACAGAGAGAGACCCTACCTT
GNB2 P1_50	ACACTCTTTCCCTACACGACGCTCTTCCGATCTATCGGCTAACTTGCACAACAGAGAGAGACCCTACCTT
GNB2 P1_51	ACACTCTTTCCCTACACGACGCTCTTCCGATCTTCGACGTCATATGCACAACAGAGAGAGACCCTACCTT
GNB2 P1_52	ACACTCTTTCCCTACACGACGCTCTTCCGATCTCGATTATGCACTGCACAACAGAGAGAGACCCTACCTT
GNB2 P1_53	ACACTCTTTCCCTACACGACGCTCTTCCGATCTGATCCGTACATTGCACAACAGAGAGAGACCCTACCTT

GNB2 P1_54	ACACTCTTTCCCTACACGACGCTCTTCCGATCTATCGATTCCGATGCACAACAGAGAGAGACCCTACCTT
GNB2 P1_55	ACACTCTTTCCCTACACGACGCTCTTCCGATCTTCGATATCGCATGCACAACAGAGAGAGACCCTACCTT
GNB2 P1_56	ACACTCTTTCCCTACACGACGCTCTTCCGATCTCGATGCTCTAATGCACAACAGAGAGAGACCCTACCTT
ZNF518B_P2	CTCGGCATTCCTGCTGAACCGCTCTTCCGATCTAGTGTGAGTATTGTGACTATGGTGCT
ZNF518B P1_66	ACACTCTTTCCCTACACGACGCTCTTCCGATCTATCGGCCTTAGTTCCTTTGGTTCAGGTAACAACATGA
ZNF518B P1_67	ACACTCTTTCCCTACACGACGCTCTTCCGATCTTCGATACGTGCTTCCTTTGGTTCAGGTAACAACATGA
ZNF518B P1_68	ACACTCTTTCCCTACACGACGCTCTTCCGATCTCGATCGCATGTTTCCTTTGGTTCAGGTAACAACATGA
ZNF518B P1_69	ACACTCTTTCCCTACACGACGCTCTTCCGATCTGATCCCGTAACTTCCTTTGGTTCAGGTAACAACATGA
ZNF518B P1_70	ACACTCTTTCCCTACACGACGCTCTTCCGATCTATCGTTGACCGTTCCTTTGGTTCAGGTAACAACATGA
ZNF518B P1_71	ACACTCTTTCCCTACACGACGCTCTTCCGATCTTCGAAGGTCTCTTCCTTTGGTTCAGGTAACAACATGA
ZNF518B P1_72	ACACTCTTTCCCTACACGACGCTCTTCCGATCTCGATGAGCCTTTTCCTTTGGTTCAGGTAACAACATGA
ZNF518B P1_73	ACACTCTTTCCCTACACGACGCTCTTCCGATCTGATCTTGTGGCTTCCTTTGGTTCAGGTAACAACATGA
ZNF518B P1_74	ACACTCTTTCCCTACACGACGCTCTTCCGATCTATCGGAGTTCCTTCCTTTGGTTCAGGTAACAACATGA
ZNF518B P1_75	ACACTCTTTCCCTACACGACGCTCTTCCGATCTTCGAAGGCTCTTTCCTTTGGTTCAGGTAACAACATGA
ZNF518B P1_76	ACACTCTTTCCCTACACGACGCTCTTCCGATCTCGATCCGATTGTTCCTTTGGTTCAGGTAACAACATGA

Supplementary Table 5. Antibodies used in the immunoblotting experiments.

Specificity	Designation	Working concentration	Specification	Source
YAP	D8h1x	1/2000	rabbit	Cell Signaling Technology
pYAP S127	D9w2i	1/2000	rabbit	Cell Signaling Technology
pErk1/2	Tyr202/204, 197G2	1/1000	rabbit	Cell Signaling Technology
pP38	Thr180/Tyr182, D3F9	1/1000	rabbit	Cell Signaling Technology
pJNK	Thr183/Tyr185, 81E11	1/1000	rabbit	Cell Signaling Technology
pS6	Ser235/236 D57.2.2E	1/2000	rabbit	Cell Signaling Technology
β-Tubulin		1/20000	rabbit	Abcam
Peroxidase-conjugated IgG		1/20000	rabbit	Jackson ImmunoResearch
